# Bone Changes in the Condylar Process of the Mandible in Computed Tomography Images and Cephalogram in a Female Patient during a Growth Spurt Treated with a Removable Functional Appliance

**DOI:** 10.1155/2020/8887182

**Published:** 2020-10-09

**Authors:** Grzegorz Rzuchowski, Marcin Mikulewicz

**Affiliations:** ^1^Dentistry “Na Biskupinie”, Orthodontic Office, Wrocław, Poland; ^2^Department of Maxillofacial Orthopedics and Orthodontics, Division of Facial Abnormalities, Wrocław Medical University, Wrocław, Poland

## Abstract

**Introduction:**

Functional treatment is the type of treatment preferred in young patients with lateral bite because it leads to simultaneous improvement of occlusion and facial profile.

**Objective:**

The aim of this study is to assess bone changes within the condylar process of the mandible and to associate them with the changes observed in the analysis of lateral cephalograms and in the patient's occlusion.

**Materials and Methods:**

Cone beam tomography of the temporomandibular joint, lateral radiogram of the skull, was performed at the beginning of treatment and after one year of therapy. Changes in cephalometric radiograms were evaluated by analyzing them and shown by making superimposition and staining layers. For the purpose of assessing bone changes within the condylar process, digital 3D solids of these processes were generated using data from computed tomography.

**Results:**

Correction towards Angle's dental class I, overjet reduction from 8 mm to 3 mm, and improvement of the patient's profile were obtained. A rotation of the occlusal plane and improvement in an ANB by 1° and in the WITS measurement by 2.7 mm were observed. A growth of the condylar processes “backwards” and “upwards” was also observed, as well as a change of their shape and volume.

**Conclusions:**

The obtained results suggest that the patient's significant improvement in occlusal conditions is due to posterior growth stimulation of the condylar processes of the mandible. The results confirm the validity of using this treatment technique in the case of growing children with complete posterior occlusion.

## 1. Introduction

Functional orthodontic treatment aims to improve the skeletal relation of the mandible to the maxilla. Intraorally, correction of II class patients involves a correction of Angle's dental class II and cuspid class II to classes I and a marked improvement with regard to overjet [1, 2]. Initially, the posterior position of the mandible is observed in the patients' facial features, with turning up of the lower lip and the distinct mentolabial sulcus [3]. If the upper incisors are tipped, the upper lip may be shortened and may not cover the teeth in the proximal section—posterior occlusion with protrusion. An analysis of the cephalogram revealed distinct deviations in the ANB angle (the angle between Downs A point, Downs B point, and Nasion) and in the WITS measurement (projection of A and B points on the occlusal plane), indicating the skeletal basis of the defect. Functional treatment is a type of orthodontic treatment which aims not only to improve the position of the mandible against the jaw but also to modify the forces of muscles on the alveolar bone. At the same time, the position of the teeth within their bone bases improves, and as the position of teeth and bones affects the external appearance of the patient, the patient's profile also improves [2, 4].

## 2. Objectives

The aim of this study was to assess bone changes in the condylar process of the mandible (based on images from computed tomography) and to relate them to the changes observed in the analysis of lateral radiographs of the skull and in the patient's occlusion.

## 3. Materials and Methods

Before starting treatment, the patient's cone beam computed tomography of the temporomandibular joint (Gendex GXCB-500 HD, USA) and the lateral radiograph of the skull (Kodak 9000 C, USA) was performed. The patient was then treated for one year with a Metzelder appliance. After a year of treatment, an identical set of comparative documentation was reperformed to assess the changes.

The research was conducted in accordance with the recommendations of the Helsinki Convention and received positive opinion of the Bioethic Commission of the Medical University in Wroclaw (No. KB-364/2014).

Two independent comparisons were made based on the following:

- Cephalometric images—changes were assessed with radiogram analysis and shown by performing superimposition along the anterior part of the cranial base and staining layers
- 3D models generated on the basis of computed tomography—bone changes in the condylar process area were assessed

3D reconstruction of the joint components based on data from CBCT enables the visualization of changes and accurate analysis of the shape and structure of the condylar process of the mandible. Generating 3D models was a multistage process:

1. Generation of the first (working) model of the mandibular ramus using ITK-SNAP software
2. Processing the working model in the Meshlab program
3. In the first stage, 3D models of the mandibular ramus were smoothed using the Laplacian smoothing algorithm in three steps. In the second stage, the 3D models of the same ramus (e.g., left) generated before and one year after the treatment are superimposed using the Align tool. This operation is performed in two stages. In the first stage, models are superimposed manually based on 4 points indicated by the researcher. These are, respectively, the top of the coronoid process, the entrance to the mandible canal, the lowest point on the notch of the mandible, and the outermost point on the condylar process. In the second stage, the perfect fit algorithm is started, which automatically corrects the initial overlap so that as much of the 3D solids as possible overlap. The difference in the size of the condyles is visible on the images. To assess these changes, a planar cut of previously imposed rami of the mandible is performed. The cutting plane is set perpendicular to the plane marked with three points: the peak of the coronoid process, the peak of the condylar process, and the lowest point of indentation of the mandible. It passes through the lowest point of the mandible indentation and is tangent to the slope of the coronoid process. This way, 3D solids of the condylar processes themselves are obtained, but with a hole at the base.
4. Closing holes created when cutting surface models and measuring the volume of solids of the processes are performed in the netfabb Basic software.
5. Superimposing and a comparative analysis of models before and after the year of treatment for showing changes are performed in CloudCompare software.

After completing all the activities, closed solids of the condylar processes are obtained that can be compared with each other.

## 4. Case Report

An 11-year-old female patient came to the orthodontic clinic concerned about her appearance. An extraoral examination showed the distinct posterior position of the mandible with the substantially turned up lower lip and deepened mentolabial sulcus. An intraoral examination and analysis of diagnostic models revealed a bilateral tendency to Angle's class II, increased overjet up to 8 mm (standard 2-3 mm), and slight crowding in the lower dental arch. The cephalometric analysis showed an ANB angle of 7.1° and a WITS of 6.8 mm. The incisors were positioned correctly, and the maxillary base angle suggests a harmonic growth. A Metzelder removable appliance was used to treat the patient.

### 4.1. The Course of Treatment

During the one-year period of treatment with the Metzelder appliance, the progress in treatment was monitored, dentition replacement was supervised, and the appliance was adapted to constantly changing occlusal conditions.

### 4.2. Evaluation of Changes on Cephalogram (Figure 1)

During the treatment with the Metzelder functional appliance, a correction of the bone base position towards the skeletal class I is expected. The key measurements, in this case, are the ANB angle and the WITS. After one year of treatment, the ANB angle was reduced by 1°. The WITS measurement was improved by 2.7 mm. Although the results still indicate the skeletal class II, correction towards Angle's dental class I was observed. The position of incisors has not changed significantly, which suggests more dentoalveolar than skeletal changes.

To illustrate these changes, superimposition of lateral cephalograms along the anterior part of the cranial base was performed as it is proved to be reliable after the age of 6 by de Coster [5], Ford [6], and most recently by Afrand et al. [7]. Cephalometric radiograms taken before the treatment—in blue—and after one year of treatment—in red—were superimposed.

**Figure 2:**
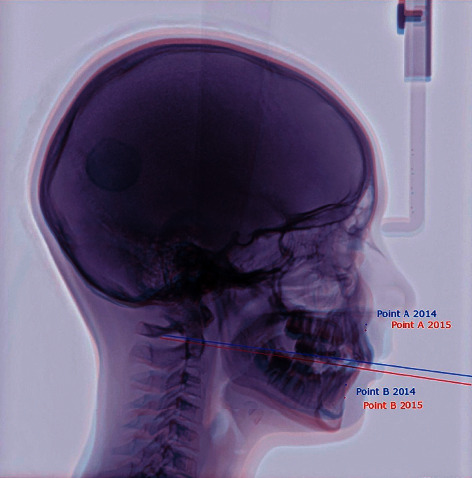
An image showing the superimposition of photographs along the anterior part of the cranial base.

Based on the analysis of Figure 2, a considerable vertical growth of the mandible and its slight anterior growth were observed. Since the growth of the mandibular ramus occurs mainly within the condylar process, it is concluded that the “backward” and “upward” growth of the process was observed in the treated patient. An important observation is also a change in the angle of inclination of the occlusal plane (this explains the improvement in the WITS value at the virtually unchanged ANB angle).

### 4.3. Evaluation of Bone Changes in the Temporomandibular Joint in the CBCT  Image

**Figure 3:**
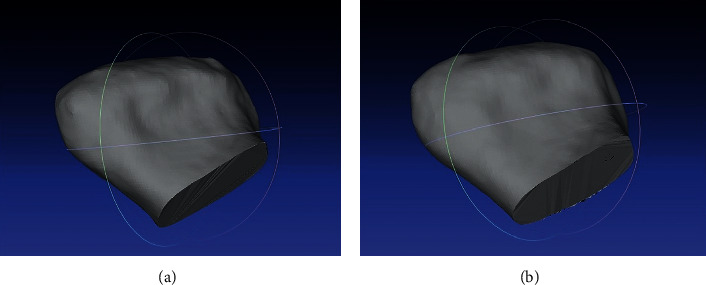
An image showing 3D models of compared mandibular condyles. (a) Mandibular head before treatment. (b) Mandibular head after one year of treatment.

**Figure 4:**
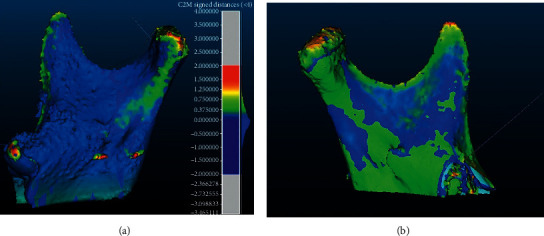
The map of changes (CloudCompare).

3D models of mandibular condyles were generated and compared using data from CBCT images (Figure 3). According to the values in Table 1, there is a considerable increase in the volume of the processes, which can then be visualized in the CloudCompare software. The colorful map shown in Figure 4 was created by superimposition of solids of the right mandibular ramus (before and after one year of treatment) and the left mandibular ramus (before and after one year of treatment). Elements that were unchanged were marked in blue, while changes up to 2 mm were shown in red. Most of them were located on the upper and posterior part of the condylar process—this is confirmed by the conclusions drawn from the analysis of lateral cephalograms regarding the growth of this element. In this particular case, the change in shape of the condylar process and its growth definitely had a positive effect on the anteroposterior relations.

## 5. Discussion

Many scientific reports suggest that treatment of posterior occlusions with removable appliances can be effective [8–12]. However, capturing changes that are responsible for improving horizontal relations is difficult and hard to investigate. This leads to the creation of numerous theories that should be thoroughly investigated. Stimulation of the posterior growth of the condylar process [8, 9] is one of the most popular in the scientific literature. However, the condylar process is the mandibular growth zone, and during a growth spurt, changes would be observed even without the applied functional treatment. Another theory concerns the articular fossa [13]. During natural growth, it moves backwards and downwards. Stopping this movement would be very useful in the treatment of posterior occlusions. In addition, it should be underlined the importance of the remodeling process within the mandibular head itself [9, 14, 15]. Studies describing changes in the shape of the condyle are most often related to injuries and treatment results using the Herbst appliance, but not exclusively [16–18]. Properly controlled resorption and bone apposition would also have a positive effect on horizontal relations in cases with skeletal class II. Researchers also noted the possibility of changing the angle between the condylar process and the mandibular ramus [19]. Such “deflection” of the entire process also would improve the relation of the mandible to the maxilla. Bones, however, are not the only area affected by a removable functional appliance. Forces are transmitted to the teeth and alveolar processes [20]; thus, the movement of entire dental arches within their bone bases is also eligible for investigation. There are at least several potential sources of changes, which is why the answer to the question what skeletal changes are caused by functional treatment in patients during growth is so difficult.

## 6. Conclusions

### 6.1. Obtained Results

1. The Angle's class I was obtained
2. Overjet reduction from 8 mm to 3 mm
3. Improving the patient's profile
4. A rotation of the occlusal plane was observed
5. Improvement of ANB by 1° and of WITS measurement by 2.7 mm

### 6.2. Conclusions Based on the Results

1. A growth of the condylar processes “backwards” and “upwards” was observed, as well as a change of their shape and volume
2. The results confirm the validity of using this treatment technique in the case of growing children with complete posterior occlusion

## Figures and Tables

**Figure 1:**
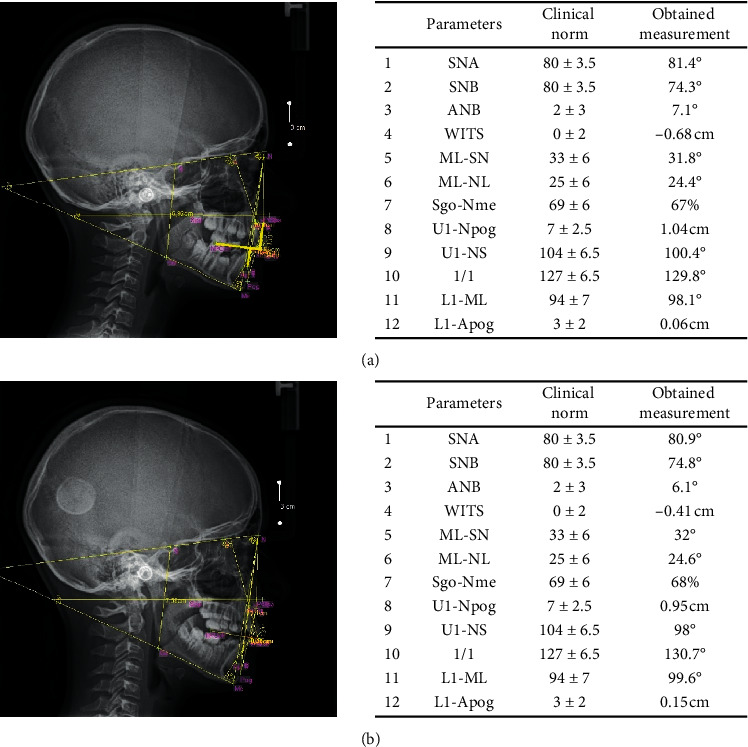
Analyzed photographs. (a) Lateral cephalogram with analysis before treatment. (b) Lateral cephalogram with analysis after a year of treatment.

**Table 1:** Results showing the changes that occurred in the mandibular condyles.

	Before treatment (cm^3^)	After treatment (cm^3^)	Change (%)
Volume of the right mandibular head	1,5595	1,7528	12
Volume of the left mandibular head	1,1946	1,3203	11

## Data Availability

All the crucial and anonymized data are part of the manuscript (like cephalometric X-rays). Some data like CBCT cannot be accessed due to privacy and law (RODO). Data such as 3D models of the mandibular ramus or mandibular head can be made available on individual request using an external server.
